# Fever Notes

**Published:** 1884-09

**Authors:** J. S. Todd

**Affiliations:** Professor of Materia Medica and Therapeutics, Atlanta Medical College, Atlanta, Ga.


### FEVER NOTES.

BY J. S. TODD, M. D.,

Professor of Materia Medica and Therapeutics, Atlanta Medical College, Atlanta, Ga.

It is not the object of this article to more than record some observations that have forced themselves on me during the past six weeks.

There is prevailing at present more or less extensively a fever, not only in Atlanta, but over the entire Northern portion of Georgia, Alabama and South Carolina, which the doctors call Typhoid—as most of them say, for want of a better name for it.

Many of the characteristics of Typhoid fever are lacking; it is "a hybrid fever, a septic fever," as my learned and venerable friend Dr. J. P. Logan, of this city, expresses it. Now I have not the time to describe it, and indeed it would be a difficult task to do so. Gulliver said of the Lilliputians,

> "Some were nicker and some were nacker,
> And some were the color of brown tobacco."

So Protean are the beginnings and course of this fever that to describe them would require a detail of almost every case separately. In some, for a few days you are sure you have nothing but an intermittent to deal with, but the impotence—utter—of cinchona and its alkaloids to control its course convinces you of your error. Again, it is a dysentery which yields to treatment, but the fever lingers. The lack of ratio between temperature, which is sometimes very high, and pulse rate often natural, makes you conceive at one time that you have the "typh-fever" of Chambers. Again, the beginning, epistaxis and diarrhœa, distended abdomen and red tongue, say Typhoid.

I have not observed that there were any eruptions, lenticular or rose colored, in any case. Constipation is the rule, and fourteen to twenty eight days the duration of the fever.

One case under Dr. Avary, which I saw with him constantly, several times daily, that of the lovable and lamented Dr. Thos. L. Raines, began as an intermittent and terminated as cerebritis. He was seen by Drs. Miller and W. F. Westmoreland in consultation. The array of attendants is a sufficient guarantee of the correctness of the treatment, which, alas, was ineffectual.

Now some cases go through the whole course of the disease without much deviation from normal heat and pulse rate.

But I will go no further into a description of the disease. I have lost one case which died in a few minutes after a profuse hemorrhage from the bowels, although there was no tympanitis, nor was there tenderness in the right iliac fossa.

The same disease raged over this section of Georgia in 1868. I was then a medical student. That spring had been characterized as has this one, by an excessive rainfall. Here in Atlanta it rained 11 inches during the month of June.

The cause of the disease is I believe water contamination, not by sewage, for in the high and healthy mountain districts it is also prevalent. Germs have been rapidly percolated through the soil, and their imbibition has given rise to the disease in question.

Quinine is not the antidote to these germs. No treatment that I have used, or that is generally used, except the one I shall mention below—and its apparent success in a few cases has prompted this article,—seems to exert any influence on the natural course of the disease, which is too often to a fatal issue.

The impotency of the usual methods: verat. viridi, to control the pulse; turpentine for its stimulant, styptic, diuretic, anti-hemorrhagic, antiseptic and carminative virtues· alcohol for the same reasons almost, and quinine on general principles, and for fashion's sake, with good nursing and alimentation, caused me to try other means.

Now, I hope and believe I am duly skeptical about the power of drugs, and make every allowance for error in diagnosis; but in six cases, which I have had under my care recently, some at the beginning, others after a week or more, showing that the fever was a continued one, and still others that had been sick for three weeks in which the treatment that I wish to record was given, and I believe, with such marked effect that I can not be silent, and must attribute the recovery to the agents used.

This is the prescription which has, I am sure, been the factor in these cases, that *cured:*

℞. Hydrarg. Corr. Chlo.................. gr. ss.
Acidi Carbolici...................... gtt. xxiv
Spt. Frumenti...................... ℥ vi.
M. Ft. Sol. Sig.—Teaspoonful every three hours, with water.

Each dose has in it the 1-96 gr. Corr. Sub., ½ drop Carbolic Acid, and 1 dr. Whisky. Opium to secure rest, and to control bowels when needed, was of course not left off. The smallness of the dose of the mercurial precluded salivation.

In no case was it necessary to continue the medicine longer than four days.

Now, gentlemen of the profession, I do not claim that I have made a discovery, but the coincident of abortion, amelioration in some cases of the disease, rapid amelioration in others, and hastened convalescence in the remainder is so remarkable that I deemed it my duty to write what I have written.

It does seem that the smallness of the doses would preclude the possibility of its acting as a germacide, but be the cause and the *modus operandi* what they may be, I simply record facts as they happened, and publish them, fully conscious of the possibility of error on my part.

The next six cases I treat may expose the fallacy of my deductions, for while I have told *the truth* about these cases, to establish *a truth* requires a large number of observations by *different* observers.

